# Efficacy of an Innovative Poly-Component Formulation in Counteracting Human Dermal Fibroblast Aging by Influencing Oxidative and Inflammatory Pathways

**DOI:** 10.3390/biomedicines12092030

**Published:** 2024-09-05

**Authors:** Francesca Rosaria Augello, Francesca Lombardi, Alessia Ciafarone, Valeria Ciummo, Serena Altamura, Maurizio Giuliani, Benedetta Cinque, Paola Palumbo

**Affiliations:** 1 Department of Life, Health and Environmental Sciences, University of L’Aquila, 67100 L’Aquila, Italy; francescarosaria.augello@univaq.it (F.R.A.); francesca.lombardi@univaq.it (F.L.); alessia.ciafarone@graduate.univaq.it (A.C.); serena.altamura@graduate.univaq.it (S.A.); maurizio.giuliani@univaq.it (M.G.); benedetta.cinque@univaq.it (B.C.); 2 Department of Innovative Technologies in Medicine and Dentistry, University “G. D’Annunzio”, 66100 Chieti, Italy; valeria.ciummo@phd.unich.it

**Keywords:** poly-component formulation, dermal fibroblast, skin aging, free radical-scavenging, anti-oxidant systems, Nrf2, pro-inflammatory cytokines, matrix metalloproteinases, AGE, NF-κB

## Abstract

Skin aging is characterized by reactive oxygen species (ROS) accumulation, principal players in triggering events associated with aging. Our recent data on the ability of an innovative poly-component formulation (KARISMA Rh Collagen^®^ FACE: K formulation) to suppress the biomolecular events associated with oxidative stress-induced aging prompted us to deepen the mechanisms underlying the observed effects on aged human dermal fibroblasts (HDFs). Here, we evaluated K’s ability to perform a direct free radical-scavenging action and modulate anti-oxidant systems by counteracting the inflammatory process in an H_2_O_2_-induced cellular senescence model. Standard methods were used to measure scavenging capacity and enzymatic anti-oxidant system activities. Nuclear factor E2-related factor 2 (Nrf2) and nuclear factor kappa-B (NF-κB) levels were analyzed by Western blot. We assessed pro-inflammatory cytokines, matrix metalloproteinases (MMPs), and advanced glycation end-products (AGEs). Our results show that K counteracted stress-induced aging in a dose-dependent manner by exerting a direct scavenging action and increasing anti-oxidant systems, such as superoxide dismutase (SOD) and catalase (CAT) up to control values. These findings could be associated with increased phospho-Nrf2 (p-Nrf2) expression, generally reduced in aged HDFs following exposure to different concentrations of K formulation. Moreover, K formulation caused a reduction of pro-inflammatory cytokines, interleukin-1β and -6, MMP-1 and -9, and AGE levels, events related to a downregulation of p-NF-κB level. The results indicate that K formulation re-established the normal physiology of HDFs by reducing p-NF-κB expression and restoring Nrf2 activation, thus supporting its efficacious reparative and regenerative action in treating skin aging.

## 1. Introduction

Skin aging is a progressive and complex process featuring numerous changes in the skin. The biological and structural changes more marked occur in the dermis where the fibroblasts, crucial players in skin homeostasis, show a limited proliferation rate, reduced biosynthetic and function activity, and substantial changes in morphology [1].

Moreover, senescent dermal fibroblasts modulate their tissue microenvironment by secreting numerous inflammatory cytokines, chemokines, growth factors, and matrix metalloproteinases (MMPs), commonly known as the senescence-associated secretory phenotype (SASP) [2]. During skin aging, the extracellular matrix (ECM), mainly produced and regulated by dermal fibroblasts, appears deleteriously disorganized due to the partial degradation of collagen and elastic fibers and can no longer provide mechanical resistance [3].

One of the main causes leading to skin homeostasis alteration underlying aging is oxidative damage, a phenomenon due to the excessive production of reactive oxygen species (ROS) and tissue incapacity of neutralizing them because the endogenous anti-oxidant system is less effective over time and saturated when these molecules are in excess [4]. The disruption in redox homeostasis of dermal fibroblasts leads to an induction of oxidative damage to essential macromolecules, therefore accelerating the skin aging process [5].

The oxidative damage can affect many skin cell functions by modulating several signaling pathways, such as mitogen-activated protein kinase (MAPK), nuclear factor kappa-B (NF-κB), transforming growth factor-β/Smad (TGF-β/Smad), and nuclear erythroid 2-related factor2/anti-oxidant response element (Nrf2/ARE) pathways [6]. NF-κB, activated downstream of MAPK, is a crucial transcription factor that up-regulates the expression levels of pro-inflammatory cytokines and matrix metalloproteinases (MMPs), responsible for ECM degradation. Therefore, modulating the NF-κB pathway can reduce oxidative stress-induced aging, as reported [7,8,9]. The TGF-β/Smad signaling is intensely involved in the regulatory process for the synthesis and deposition of collagen and its degradation and then in maintaining ECM homeostasis. Increased ROS levels negatively affect TGF-β signaling, leading to the reduction of type I collagen synthesis [10]. Nrf2 is a transcription factor key in the defense system against oxidative stress since it is involved in the up-regulation of anti-oxidant enzymes in the cellular response to oxidative stress [11,12]. For this reason, the Nrf2 pathway has stimulated profound interest, suggesting its activation as a potential therapeutic target to protect against oxidative stress [13]. Anti-oxidant enzymes, such as catalase (CAT), superoxide dismutase (SOD), and glutathione peroxidase (GSH-Px), represent the anti-oxidant defense system in the skin to cope with the physiological level of oxidation. However, the enzymatic and non-enzymatic anti-oxidant system weakens with age, making aged skin more susceptible to external stimuli, such as UV radiation, smoking, and pollution [14].

Moreover, a group of heterogeneous molecules known as advanced glycation end-products (AGEs) plays a crucial role in human skin aging, obtained through a non-enzymatic reaction between reducing sugars and free amino groups of proteins, lipids, or nucleic acids. AGEs alter deposition, physicochemical properties, and organization of dermal ECM components. Glycated fibers lose elasticity, become rigid, and have reduced regenerative ability, leading to skin damage [15]. Therefore, high levels of AGEs in the skin can impair skin function, contributing to the skin aging phenotype, oxidative stress, and inflammation. Given the above, using innovative strategies that can restore and sustain the ability of dermal fibroblasts to counteract the disturbance of redox balance and that can decrease glycation or repress AGE synthesis would be a promising approach for hindering age-related skin problems and slowing the progression of aging and inflammation [16].

Given the growing aging of the population and the significant social relevance associated with aging, it is of great social and scientific interest to enhance the development of novel and efficacious anti-aging products. The most effective approaches to counteract the appearance of visible signs of skin aging include topical agents, energy-based procedures, and dermal fillers able to provide practical components or technologies that, acting on the epidermis and dermis, can modulate the main mechanisms associated with aging and restore the molecular features of the skin with clinical efficacy [17].

Several molecules are used as topical agents for the improvement of skin aging signs, such as anti-oxidants (ascorbic acid) [18], hydroxy acids (glycolic or lactic acid) [19], and retinoids, which are still considered the gold standard among all these products [20]. Other popular anti-aging procedures, common for dermal rejuvenation, are those that involve energy-based devices, such as lasers, high-intensity focused ultrasound (HFU), and radiofrequency (RF) devices. These devices are based on the thermal energy delivery to the reticular dermis and subcutaneous compartment, so tissue contraction and neocollagenesis stimulation are achieved, leading to improvement in skin laxity and rhytides for skin rejuvenation and resurfacing [21,22,23].

There is growing interest in using injectables to fill wrinkles and replace soft-tissue volume in aged skin, consequently improving skin texture. Moreover, recent evidence has displayed that fillers can improve signs of skin aging by structurally supporting the ECM and stimulating the fibroblast functions in aged human skin, restoring cutaneous homeostasis [24,25,26,27,28]. The recent market availability of an innovative injective poly-component formulation (KARISMA Rh Collagen^®^ FACE, K) consisting of non-crosslinked high-molecular-weight hyaluronic acid (HMW-HA, MW 1500–3000 kDa), a human recombinant polypeptide of collagen-1 alpha chain and carboxymethyl cellulose (CMC), hereinafter indicated as “K formulation” caught our scientific interest. The innovative nature of K formulation is due, in particular, to the presence of human recombinant polypeptide of collagen-1 alpha chain, a molecular configuration of the collagen not reported in other dermal fillers. A patented methodology has been applied to generate it using transgenic silkworms to stimulate the alpha helix filament synthesis within their cocoons. K formulation is an injectable and absorbable medical device; it is a bio-restorative soft filler able to naturally restore pre-aging conditions by carrying out its action on the renewal of the extracellular matrix. This innovative formulation is recommended for those who wish to correct facial skin imperfections due to skin aging and/or loss of volume, obtaining a medium-term improvement. In our recent in vitro study [29], we reported the ability of K formulation to suppress the biomolecular events associated with oxidative stress-induced dermal fibroblast aging. In particular, our findings showed that the poly-component formulation improved in vitro the adult fibroblasts’ biological activities, including proliferation, migration, and contraction ability, as well as counteracted reactive oxygen species production in aged fibroblasts. More recently, Di Rosa et al. [30] evaluated the effectiveness of the K formulation on 100 subjects with different skin conditions, confirming the product’s effectiveness in skin regeneration and aesthetic improvement. The exposure to K formulation reduced the skin roughness values in the group of people with skin aging of different degrees, in the smoker subjects affected by skin aging, and in patients with facial scarring. Furthermore, the authors also recorded a high satisfaction level of the patients by psychometric tests.

Based on these previous in vitro and in vivo studies, the present study aims to deepen the mechanisms underlying the K formulation’s anti-aging effects. We first focus on the formulation’s potential to exert a direct scavenging action. Then, the influence on the biomolecular pathways related to oxidative stress and the inflammatory profile associated with dermal fibroblast aging was evaluated.

## 2. Materials and Methods

### 2.1. Preparation of K Poly-Component Formulation for Cell Treatments

KARISMA Rh Collagen^®^ FACE, a bio-restorative formulation (K formulation) (kindly provided by Taumedika Srl, Rome, Italy), composed of 2 mg/mL high-molecular-weight hyaluronic acid (HMW-HA), 50 µg/mL human recombinant collagen α1 chain, and 40 mg/mL carboxymethylcellulose (CMC). For cell exposure, the “extraction dilution method” described by the UNI EN ISO 10993 regulation [31] was used to prepare the K formulation. Briefly, the extraction protocol was carried out in an extraction medium that included complete medium DMEM, supplemented with 10% of fetal bovine serum (FBS), 100 U/mL penicillin, 100 mg/mL streptomycin, and 2 mm glutamine at 37 °C ± 1 for 24 h, by continuous agitation. Subsequently, for all the below described in vitro cell treatments, K formulation was serially diluted and used at three different concentrations (0.5, 2, and 5% *v*/*v*, final concentration) based on our previous data on the effect of K formulation on cell viability evaluated by Trypan blue test [29].

### 2.2. Cell Systems

**Figure 1:**
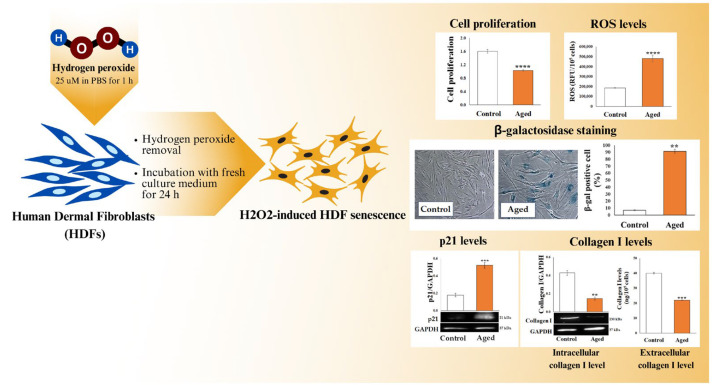
One-step model of H_2_O_2_-induced HDF senescence. Multi-parametric evaluation of H_2_O_2_-induced senescence in HDFs. The results of the cell proliferation, analyzed as cell confluence through the IncuCyte^®^ system, and ROS levels, assayed using the DCFH-DA, are shown as the mean ± SEM. Cellular aging, determined by β-galactosidase staining, was expressed as the percentage of positively blue-stained HDFs, and representative images are shown (20× magnification). The p21, intracellular, and extracellular collagen I levels were analyzed from three independent experiments (mean ± SEM). All shown data are relative to 72 h from treatment. For the comparative analysis of the data, Student’s unpaired *t*-test was used (** *p* < 0.01, *** *p* < 0.001, **** *p* < 0.0001 vs. control)”. This image was created with www.canva.com, accessed on 15 July 2024.

Adult normal primary human dermal fibroblast (NHDFs, Adult CC-2511) cell line was acquired from Lonza/Cell Applications (Basel, Switzerland) and routinely cultured in a complete medium. Cells were maintained in a humidified atmosphere of 95% air and 5% CO_2_ at 37 °C. All reagents and consumables were obtained from Euro Clone (West York, UK). Until the 15^th^ passage, the cells were used for the experiments. To study the effect of the K formulation, we used a primary human dermal fibroblast line, a model largely used to test innovative molecules and formulations in preventing or counteracting the skin aging process [32,33]. For the induction of senescence, a previously described model of hydrogen peroxide (H_2_O_2_)—induced senescence of HDFs was utilized and slightly modified [34]. Briefly, HDFs (plated at 7000 cells/cm^2^) at 70% confluence were exposed to 25 µM H_2_O_2_ (AppliChem GmbH, Darmstadt, Germany) in PBS for 1 h in a humidified atmosphere at 37 °C with 5% CO_2_, and then, the H_2_O_2_ solution was substituted with the complete medium for 24 h. The adopted model indicated as a one-step model, allows us to obtain senescent cells with the classic characteristics that make them suitable for evaluating potential anti-aging effects [29]. To ascertain the full achievement of the cellular aging status through the one-step model, the following parameters were regularly monitored: cell proliferation, intracellular ROS levels, β-galactosidase activity, p21 expression level, and collagen level (Figure 1). The cell proliferation was analyzed as cell confluence using the IncuCyte^®^ Live Cell Imager system (Essen BioSciences, Inc., Ann Arbor, MI, USA) for real-time evaluation of cell confluence. Briefly, cells, plated in a 96-multiwell culture plate 2.5 × 10^3^ cells/well, allowed to attach overnight, were treated with the different concentrations of K formulation. Culture plates were sited into the IncuCyte^®^ Live Cell imager, and images acquired with phase contrast channel were taken every 6 h from 0 to 72 h post-exposure. Two image sets were captured from several points of the well using a 10× objective lens. All the exposure conditions were run in triplicates. The cell proliferation (phase area confluence normalized to T0) was quantified and analyzed by IncuCyte ZOOM™ software (2020b, Essen BioSciences, Inc.). Intracellular ROS levels were evaluated using a dichloro-dihydro-fluorescein diacetate (DCFH-DA) probe (Immunological Sciences, Rome, Italy). According to the manufacturer’s instructions, β-galactosidase activity was assessed using a kit by Cell Signaling Technology (Danvers, MA, USA). p21 and collagen I levels were evaluated using a Western blot assay. Extracellular collagen I levels were assayed in the cell supernatants by the human type I collagen ELISA kit (Immunological Sciences). Subsequently, different concentrations of K formulation were added to the cells (aged HDFs). We considered the adult HDFs (Control) as controls.

### 2.3. DPPH Radical-Scavenging Activity Assay

DPPH is used to evaluate the radical-scavenging activity of substances in a cell-free system; it is a stable free radical with an absorption wavelength of 517 nm. A compound with anti-oxidant activity can provide an electron or hydrogen atom to DPPH (purple) and produce a reduced form of DPPH (yellow). The rate of color shift from purple to yellow is directly proportional to the radical-scavenging activity of the compound. Here, DPPH in methanol (150 µM; 180 µL) was added at three different percentages of K formulation in methanol (0–0.5–2 and 5%; 20 µL) into 96-well plates and incubated for 30 min in the dark at room temperature. The absorbance was read at 517 nm by a spectrophotometer (Eppendorf, Hamburg, Germany). Vitamin C (ascorbic acid) was used as a positive control at 300 µM. The % of radical-scavenging activity was determined as follows:

%RSA = ((A_blank_ − A_sample_)/A_blank_) × 100

where A_blank_ and A_sample_ are the absorbance values of the blank and sample, respectively.

### 2.4. Superoxide Dismutase (SOD) Activity

SOD activity was evaluated by a SOD assay kit (Cayman Chemical Company, Ann Arbor, MI, USA) in the cell lysates according to the manufacturer’s instructions. In brief, aged HDFs were exposed to K at 0.5, 2, or 5% for 72 h. Following treatments, the cells were harvested, lysed, and centrifugated at 17,949× *g* for 20 min. Then, SOD activity is evaluated by measuring the superoxide radicals generated by xanthine oxidase and hypoxanthine and detected by tetrazolium salt. The absorbance was measured by spectrophotometric reading (BioRad, Hercules, CA, USA) at 450 nm. The values were interpolated with a standard curve with known concentrations of enzyme. The data, normalized for total protein content evaluated by DC Protein Assay (BioRad), were expressed as units of SOD activity/mg protein.

### 2.5. Catalase (CAT) Activity

CAT activity in the cell lysates was quantified by a CAT assay kit (Cayman Chemical Company), according to the manufacturer’s recommendations. To eliminate cell debris, after treatments, the cells were lysed and centrifugated at 17,949× *g* for 20 min. The cell lysates were evaluated for protein content with a DC Protein Assay (BioRad). Then, CAT activity was measured by detecting spectrophotometrically at 540 nm the formaldehyde produced by the reaction mix. The values were interpolated with a standard curve with known concentrations of formaldehyde. Obtained data were normalized for total protein and expressed as units of CAT activity/mg protein.

### 2.6. Western Blot Analysis

For protein analysis through Western blot, after treatments, the cell pellets were harvested, washed in PBS, and then lysed in RIPA Lysis Buffer (Merck KGaA, Darmstadt, Germany) in the presence of 100 mM protease inhibitor cocktail (Sigma-Aldrich, St. Louis, MO, USA). Then, the supernatant was collected after centrifugation at 17,949× *g*, eliminating cell debris, and the total protein content was assessed using DC Protein Assay (BioRad, Hercules, CA, USA). Equal amounts of protein solutions (25 µg/well) were separated by 10% SDS-polyacrylamide gel electrophoresis and transferred onto 0.45 µm nitrocellulose membrane sheets (BioRad) for 1 h at 4 °C. After blocking of the specific sites with 5% nonfat dry milk, the membranes were incubated overnight at 4 °C with mouse monoclonal antibody anti-p21 1:1000 (Cell Signaling Technology, Danvers, MA, USA), rabbit polyclonal antibody anti-COL1A1 1:1000 (Boster Biological Technology, Pleasanton, CA, USA), rabbit monoclonal antibody anti-phospho-Nrf2 (phospho-S40) 1:2000 (Abcam, Cambridge, UK), rabbit monoclonal antibody anti-phospho-NF-κB (phospho-S536) 1:1000 (Cell Signaling Technology), rabbit monoclonal antibody anti-NF-κB 1:1000 (Cell Signaling Technology), and mouse monoclonal antibody anti-GAPDH 1:2000 (Immunological Sciences, Rome, Italy). The membranes were then incubated with secondary antibodies: peroxidase-conjugated goat anti-rabbit or rabbit anti-mouse IgG 1:5000 (Immunological Sciences). After washing, the densities of immunoreactive bands visualized using chemiluminescence reagent (ECL, Amersham Pharmacia Biotech, Buckinghamshire, UK) were quantified by the chemiluminescence documentation system ALLIANCE (UVITEC, Cambridge, UK).

### 2.7. Immunofluorescence Assay for Nrf2 

HDFs were grown on coverslips in a 12-well plate (seeded at 7000 cells/cm^2^), and aged cells obtained by a one-step model were treated with or without K at 0.5, 2, or 5% for 72 h. At the end of treatment, the cells on the coverslips, after washing with PBS and formaldehyde fixation (4% for 20 min), were permeabilized with 0.1% Triton X-100 (Sigma-Aldrich) for 5 min and blocked with 3% BSA (Sigma-Aldrich) for 20 min at room temperature. Afterward, coverslips were incubated for 18 h at 4 °C with rabbit monoclonal antibody anti-phospho-Nrf2 (phospho-S40) 1:250 (Abcam). FITC conjugated goat anti-rabbit polyclonal IgG secondary antibody (Millipore EMD, Darmstadt, Germany) was used at 1:1000 for 1 h at room temperature. Then, the coverslips were stained for 45 min with TRITC-labeled phalloidin (Sigma-Aldrich) at room temperature. Antifade Mounting Medium with added DAPI (Vector Laboratories, Inc., Burlingame, CA, USA) was used to counterstain nuclei and to mount the coverslips, which were then examined at 100× magnifications by fluorescence microscopy (Eclipse 50i, Nikon, Tokyo, Japan).

### 2.8. Enzyme-Linked Immunostaining Assay for Interleukin Measurement

The supernatant was analyzed for IL-1β and IL-6 content according to the company’s protocol using a human IL-1β ELISA kit (Sigma-Aldrich, Saint Louis, MO, USA) or a human IL-6 ELISA kit (Sigma-Aldrich). In brief, after treatments, the culture media were collected and centrifuged at 1000× *g* for 15 min to clarify them by cellular debris/dead cells. The IL-β1 and IL-6 concentrations were then measured with an ELISA kit. The obtained values were normalized for cell number and expressed as pg/10^5^ cells.

### 2.9. Total RNA Extraction and Quantitative Real-Time PCR (qPCR)

Gene expression analysis of MMP-1 and MMP-9 was performed in control and aged HDFs treated with K formulation at 0.5, 2, or 5% by real-time RT-PCR using a ViiA7 sequence detection system (Applied Biosystems, Foster City, CA, USA). Total RNA was extracted by the RNeasy Mini Kit (QIAGEN, Hilden, Germany) and quantified by spectrophotometry. A total of 1 μg of RNA was reverse transcribed using a mixture of random primers given below. An amount of 0.5 µg of each cDNA was used to perform the real-time PCR. Real-time quantitative RT-PCR analysis was carried out using SYBR Green dye detection (Thermo Fisher Scientific, Waltham, MA, USA). Forward and reverse primers, purchased from Integrated DNA Technologies (Coralville, IA, USA), were used at a concentration of 1 µM (MMP-1, MMP-9, GAPDH), and their sequences are reported in Table 1. The fold-change quantification of target genes was measured with the 2 ^−DDCt^ method. The experiments were performed twice, and the samples were run in triplicate.

### 2.10. Advanced Glycation End-Product (AGE) ELISA Kit

Advanced glycation end-product (AGE) levels were measured using the AGE Elisa Kit (Immunological Sciences, Rome, Italy) in the cell lysates. Briefly, untreated and treated, control and aged HDFs were collected, lysed, and centrifugated at 17,949× *g* for 20 min. AGE levels assessed using an ELISA kit were normalized for cell number and then expressed as ng/10^5^ cells.

### 2.11. Statistical Analysis

All data were evaluated by GraphPad Prism version 6.01 (GraphPad Software, San Diego, CA, USA). For comparison between the two means, the Student’s unpaired *t*-test was used. For comparison of the mean values among groups, one-way ANOVA, followed by Tukey or Dunnett post hoc test, was used. Data were expressed as mean ± SD or mean ± SEM as reported in figure legends. The *p* values were considered statistically significant when lower than 0.05.

## 3. Results

### 3.1. K Formulation Neutralized Oxidative Damage by Radical-Scavenging Capacity

Our recent data have reported the ability of K formulation to improve the proliferation, migration, and contractile activity in H_2_O_2_-induced human dermal fibroblast senescence model and their biosynthetic activity, showing the potential to reduce ROS levels in the same model [29]. To evaluate this anti-oxidant action, the direct scavenging ability of K was assessed using the conventional DPPH assay.

**Figure 2:**
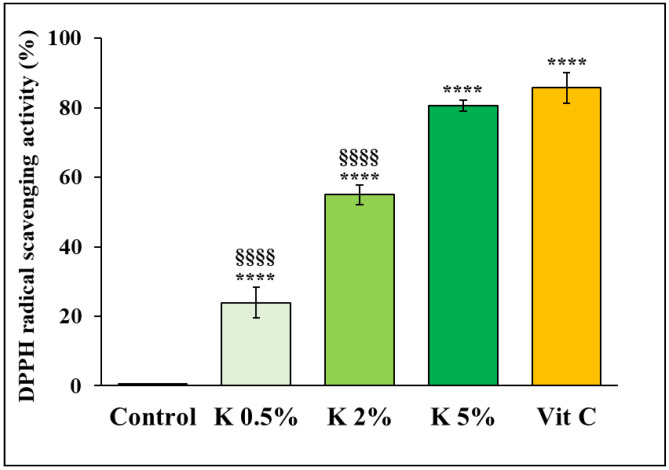
Free radical-scavenging activity of K formulation. The free radical-scavenging activity of K formulation was evaluated using the DPPH assay. Vitamin C (ascorbic acid; 300 µM) was used as a positive control. The results are expressed as mean ± SD (*n* = 4). For comparative analysis of groups of data, one-way ANOVA followed by Tukey’s post hoc test was used (**** *p* < 0.0001 vs. control, §§§§ *p* < 0.0001 vs. Vit C).

Figure 2 shows the K formulation’s DPPH radical-scavenging activity, used at three different percentages (0.5, 2, and 5%) and 300 µM vitamin C (ascorbic acid) as a positive control. The results indicated that the free radical inhibition activity increased in a dose-dependent manner. Notably, the activity of the K formulation at 5% was not significantly different from the positive control.

### 3.2. K Formulation Prevented Aging-Associated Oxidative Stress by Enhancing Cellular Anti-Oxidant Enzyme Activity

**Figure 3:**
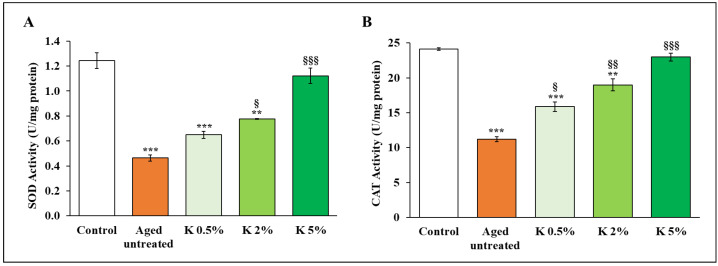
Effect of K formulation on SOD and CAT activities in aged HDFs. The activities of SOD (**A**) and CAT (**B**) were evaluated in aged HDFs treated whit increasing concentrations of K for 72 h, using an assay kit. Results are relative to mean values ± SEM of two experiments performed in duplicate. For comparative analysis of groups of data, the one-way ANOVA with Tukey’s post hoc test was used (** *p* < 0.01, *** *p* < 0.001 vs. control; § *p* < 0.05, §§ *p* < 0.01, §§§ *p* < 0.001 vs. aged untreated).

As the second step, we evaluated the ability of K formulation to enhance the activity of the enzymatic anti-oxidant defense system mainly attributed to SOD and CAT enzymes. Indeed, often, the increased intracellular ROS levels in aged cells are generally accompanied by lower SOD and CAT activities [35]. As expected, the SOD and CAT activities were markedly reduced in the H_2_O_2_-induced senescent HDFs with respect to the relative control, but when the K formulation was added at different concentrations (0.5, 2, and 5%) on the senescent HDFs, for 72 h, both enzymatic activities significantly augmented compared with aged untreated (Figure 3). Of note, the higher concentration of the K restored both the enzymatic activities up to control values, with no significant difference with control HDFs.

### 3.3. Effect of K Formulation on Phospho-Nrf2 Expression in Aged HDFs

**Figure 4:**
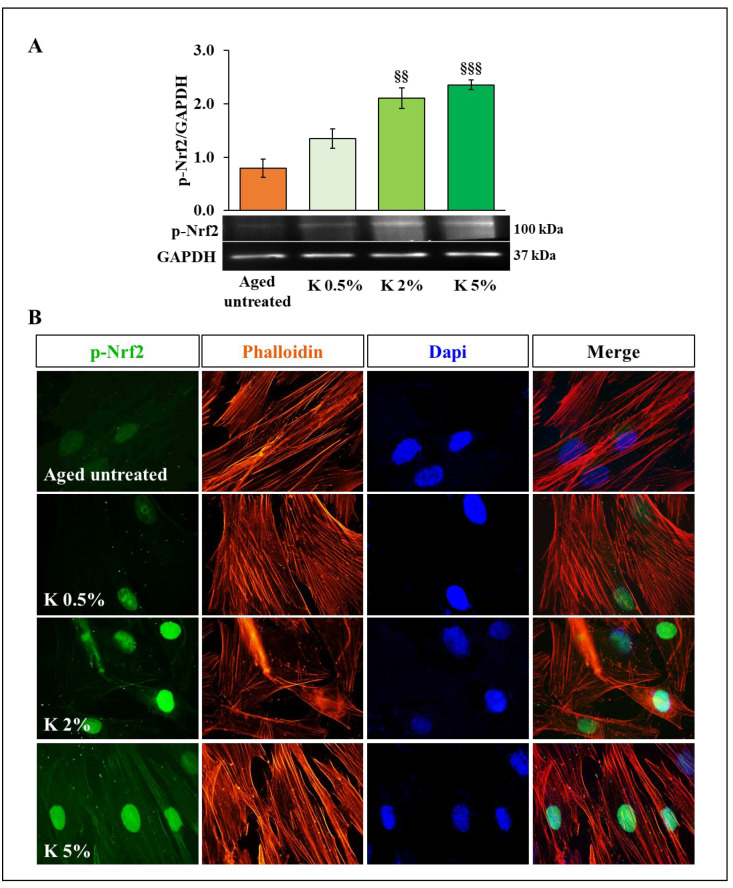
Effect of K formulation on p-Nrf2 expression in aged HDFs. (**A**) p-Nrf2 immunoblotting assay was performed on aged HDFs stimulated for 72 h with K formulation at different percentages. The values obtained by densitometric analysis were normalized vs. GAPDH protein. The results from three independent experiments are shown as the mean ± SEM. For comparative analysis of groups of data, one-way ANOVA followed by Dunnett’s post hoc test was used (§§ *p* < 0.01, §§§ *p* < 0.001 vs. aged untreated). (**B**) Representative immunofluorescence images of HDFs stained with anti-*p*-Nrf2 antibody (green) and with TRITC-phalloidin (red) to reveal F-actin. Nuclei were counterstained with DAPI (blue). All images were acquired at 100× magnification.

To clarify the mechanism by which K formulation increased anti-oxidant defense capacity in the aged HDFs through the improvement of SOD and CAT activities, we evaluated the potential involvement of Nrf2. This evaluation was not reported for the control group since this sample showed the detectable activities of SOD and catalase, indicating a well-functioning redox equilibrium. As expected, in aged control, the phospho-Nrf2 (p-Nrf2) expression appeared faintly, while the treatment with increasing concentrations of K for 72 h caused a dose-dependent raise of the p-Nrf2 levels in aged HDFs (Figure 4A). Consistent with the Western blotting results, we observed that the nuclear immunolocalization of the p-Nrf2, undetectable in aged untreated HDFs, augmented gradually with increasing concentrations of K formulation (Figure 4B).

### 3.4. Effect of K Formulation on Oxidative Stress-Induced NF-κB Pathway 

**Figure 5:**
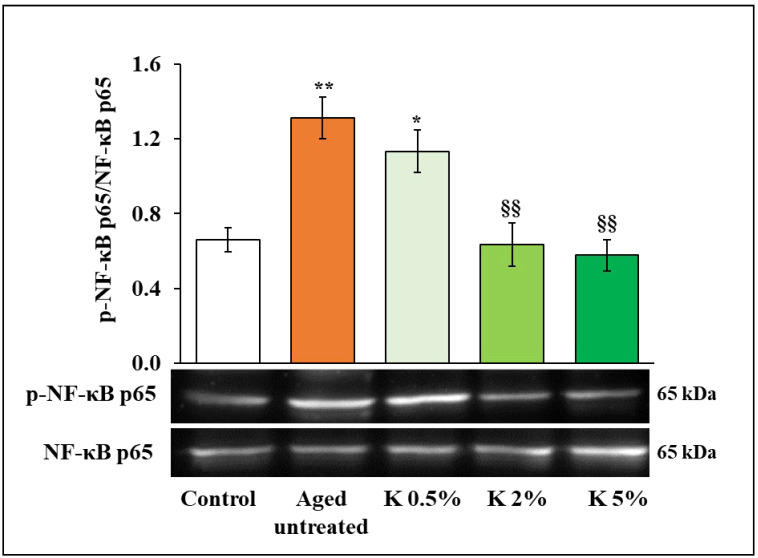
Effect of K formulation on p-NF-κB expression in aged HDFs. Immunoblotting assay for p-NF-κB was performed on control and aged HDFs treated with increasing concentrations of K for 72 h. The values obtained by densitometric analysis were normalized vs. NF-κB. The results from three independent experiments are shown as the mean ± SEM. For comparative analysis of groups of data, one-way ANOVA followed by Tukey’s post hoc test was used (* *p* < 0.05, ** *p* < 0.01 vs. control; §§ *p* < 0.01 vs. aged untreated).

Establishing an inflammatory dermal microenvironment is one of the major characteristics of aged skin. Indeed, in aged skin, high levels of ROS cause the activation of NF-κB, which leads to an increase in pro-inflammatory cytokines and MMP expression, responsible for ECM degradation [36]. Then, we evaluated the effects of K treatment on the NF-κB activation in H_2_O_2_-induced senescent HDFs by Western blot. As shown in Figure 5, H_2_O_2_ induced a significant increase in the ratio of phospho-NF-κB (p-NF-κB) and NF-κB, while the addition of K formulation could reverse this effect in a dose-dependent way. Of note, at 2 and 5% K concentration, the NF-κB activation in aged cells returned to the control cell level.

**Figure 6:**
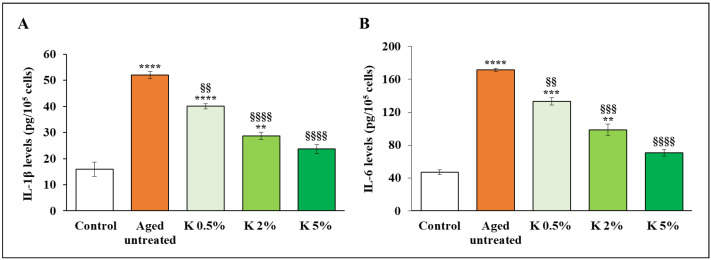
Effect of K formulation on pro-inflammatory cytokines in aged HDFs. (**A**) IL-1β and (**B**) IL-6 levels were evaluated by an ELISA kit in cell supernatants of control and aged HDFs untreated and treated with increasing concentrations of K for 72 h. The results from three independent experiments are shown as the mean ± SEM. For comparative analysis of groups of data, one-way ANOVA followed by Tukey’s post hoc test was used (** *p* < 0.01, *** *p* < 0.001, **** *p* < 0.0001 vs. control; §§ *p* < 0.01, §§§ *p* < 0.001, §§§§ *p* < 0.0001 vs. aged untreated).

The secretion of two crucial pro-inflammatory cytokines, IL-1β and IL-6, was also evaluated after K formulation treatment. As shown in Figure 6A,B, the levels of IL-1β and IL-6 in aged cells were significantly increased by about 3- and 4-fold than relative controls. The addition of K formulation led to a significant decrease of both cytokines in a concentration-dependent manner in aged HDFs; notably, in the presence of 5% K, the cytokine profile was not significantly different from the relative controls.

**Figure 7:**
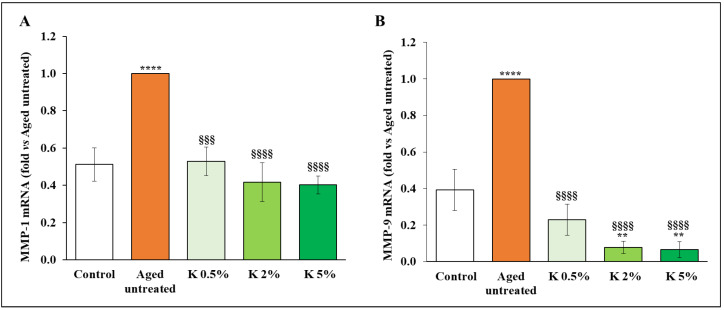
Effect of K formulation on MMP-1 and -9 expression in aged HDFs. The SYBRGreen Real-Time PCR analysis of (**A**) MMP-1 and (**B**) MMP-9 genes was performed on control and aged HDFs untreated and treated with increasing concentrations of K for 72 h. mRNA levels were relative to the amount of GAPDH mRNA. Results relative to one representative out of two independent experiments are expressed as mean ± SD (*n* = 3). For comparative analysis of data groups, one-way ANOVA followed by Tukey’s post hoc test was used (**** *p* < 0.0001, ** *p* < 0.01 vs. control; §§§ *p* < 0.001, §§§§ *p* < 0.0001 vs. aged untreated).

We also investigated the effect of K on MMP levels, which are strongly regulated by NF-κB, and crucial factors in promoting the deleterious disorganization of the dermal ECM in aged skin, consequently causing a loss of elasticity. In particular, the increased cleavage of collagen and elastin fibrils by MMP-1 and -9 appears to be the major aspect of skin aging processes [37]. For this reason, we analyzed the mRNA levels of MMP-1 and -9 genes by RT-qPCR, confirming that H_2_O_2_ exposure increased the expression levels of both MMPs in dermal fibroblasts with respect to control by doubling them (Figure 7A,B). Elevation of MMP-1 and -9 expression was significantly lowered by K formulation treatment, and this effect was concentration-dependent, with the higher concentration able to restore the mRNA levels to control levels.

### 3.5. Antiglycation Assessment of K Formulation

**Figure 8:**
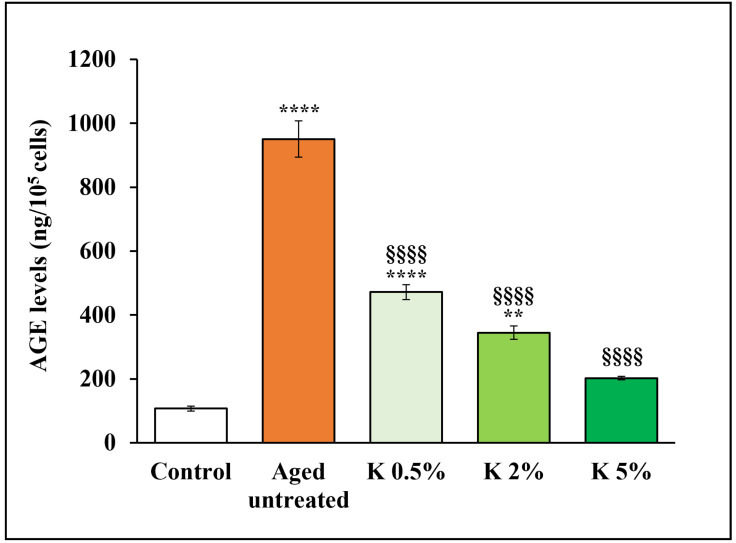
Effect of K formulation on AGE levels in aged HDFs. Effect of K formulation on AGE levels in aged HDFs. AGE levels in control and aged HDFs treated with K formulation for 72 h were assayed in cell lysates by AGE ELISA kit. Results are relative to mean values ± SEM of three experiments performed in duplicate. For comparative analysis of data groups, one-way followed by Tukey’s post hoc test was used (** *p* < 0.01, **** *p* < 0.0001 vs. control; §§§§ *p* < 0.0001 vs. aged untreated).

The formation of AGEs is a crucial process involved in skin aging, being able to alter skin homeostasis and injure the dermal extracellular matrix [15]. In addition, oxidative stress is considered fundamental in forming endogenous AGEs, making oxidative radicals the most critical participants in glycation reactions [16]. Thus, we evaluated the effect of K formulation on the AGE production by aged HDFs. As reported in Figure 8, AGE levels in aged HDFs were 9-fold higher than control HDFs, indicating that glycation was induced in our model of oxidative stress-induced senescence, as expected. Of note, when the aged HDFs were exposed to K formulation, the levels of AGEs significantly decreased in a concentration-dependent manner compared to that of the aged untreated fibroblasts, suggesting that the formulation might either decrease the generation rate of AGEs or contribute to their clearance. Of note, the AGE levels at K 5% concentration did not appear significantly different from that of the control.

## 4. Discussion

The alteration of skin homeostasis appears as a peculiar feature of skin aging due to an imbalance in the production and degradation of the extracellular matrix components, the replicative senescence, and the marked inflammatory profile. These characteristics are closely associated with oxidative stress, which causes an accumulation of ROS. Although the molecular mechanisms of skin aging are intricate, high levels of ROS, apart from intrinsic or extrinsic aging, can regulate several significant signals, leading to cellular damage and weakening of cell function and thus causing cellular senescence [4,36]. Of note, since oxidative stress, inflammation, and aging are closely associated and ROS play a crucial role in exacerbating both oxidative stress and inflammation, the use of compounds that perform anti-oxidant actions could represent a practical approach to alleviating the damage caused by ROS in the skin, thus slowing down or counteracting the skin aging process.

The present study aimed to verify the hypothesis that the regenerative and bio-revitalizing actions of an innovative injective poly-component K formulation (KARISMA Rh Collagen^®^ FACE) previously demonstrated both in vitro and in vivo [29,30] may be due to a potential direct scavenging action as well as to its ability to interfere with fibroblast aging-related oxidative and inflammatory pathways.

Several studies have shown that injectables represent one of the most effective approaches to hinder or counteract the critical signs of skin aging [38,39,40]. The fillers can enhance the structural support of the ECM, restore the contractile capacity of aged dermal fibroblasts, induce collagen synthesis, and increase fibroblast proliferation [39,41,42]. HA is the most used component in injectable formulations for its biocompatibility and ease of use, often used with other ingredients such as amino acids [43]. Shin et al. demonstrated that an EGF-containing HA filler induced types I and III collagen synthesis and downregulated the expression of MMP-1, -3, and -9 in a photoaged mouse model [44]. A recent study assessed the anti-aging properties of an HA matrix isolated and purified from the rooster comb. The evaluation of the HA matrix’s biological activity showed regenerative properties in keratinocytes and fibroblasts, as well as anti-aging, moisturizing, and anti-oxidant effects [45]. Moreover, other studies have revealed the capability of some polymers, such as poly-L-lactic acid, polycaprolactone, and polynucleotide, to enhance fibroblast functions and induce neocollagenesis; therefore, these polymers have been included in the injectable formulation [39,46]. In this regard, Oh et al. demonstrated that a Poly-D, L-lactic acid (PDLLA) filler increased Nrf2 expression in macrophages, resulting in increased M2 polarization and IL-10 expression in senescent macrophages. The increased IL-10 expression reduced adipose-derived stem cell (ASC) senescence and enhanced their ability to release TGF-β and FGF2. This, in turn, led to increased fibroblast proliferation and collagen synthesis, resulting in rejuvenation in aged skin [26]. Consisting with our previous results showing the capacity of K formulation to reduce ROS and malondialdehyde levels in a model of stress-induced HDF aging, we investigated some potential mechanisms underlying the effects of K against oxidative imbalance in aged cells. Design experiments to evaluate direct anti-oxidant activity show evidence of K formulation’s relevant and concentration-dependent free radical-scavenging activity, as determined by the DPPH assay. This effect could be attributed to hyaluronic acid (HA), one of the components of the K formulation. In this regard, it is helpful to underline that, recently, HA has been suggested to have the potential of a natural anti-oxidant, with a relevant scavenging capacity and reducing power and chelating activity for ferrous ions [47,48].

On the other hand, we show evidence that K formulation addition also led to a concentration-dependent increase of SOD and CAT, the major enzymes involved in the body’s defense systems against oxidative stress [14,49]. Stimulating these anti-oxidant systems after treatment with K formulation was associated with an increased expression of phosphorylated Nrf2. The maintaining of redox balance presumed the involvement of the Nrf2-mediated signaling, being Nfr2 the transcriptional master regulator of multiple anti-oxidant enzyme genes, such as SOD and CAT [50,51], whose promoters share the same anti-oxidant response element (ARE) sequence of NF-κB, another crucial transcription factor involved in the inflammatory and secretory profile associated with senescence. Of note, our findings also showed that K formulation significantly downregulated p-NF-κB in aged HDFs, thus leading to decreased secretion of pro-inflammatory cytokines, such as IL-1β and IL-6, as well as MMP-1 and -9, all considered typical SASP components of aged HDFs. In addition, K formulation decreased, in our experimental aging model, the accumulation of AGEs, which formation is known to be strongly associated with cellular aging and induced by inflammatory processes and oxidative stress mechanisms [16,52]. These results suggest that K formulation could indirectly counteract the glycation process associated with oxidative damage due to its demonstrated anti-oxidant properties, even if we cannot exclude other mechanisms, including direct inhibition of the glycation process or the stimulation of the AGEs’ clearance.

Cellular oxidative stress and inflammation responses result from a refined crosstalk mechanism between NF-κB and Nrf2 pathways. The predominance of the former over the latter will favor inflammation and oxidative stress; on the contrary, the prevalence of the Nrf2 pathway will perform an anti-inflammatory action and restore redox balance, playing a protective role for cells. Studies show that the heme-oxygenase gene 1 (HO-1), activated by Nrf2 signaling, is central in mediating this crosstalk. HO-1′s end-products can prevent the nuclear translocation of NF-κB, turning off its pathway [53,54,55]. The cellular overproduction of ROS, or the impaired anti-oxidant systems, causes the increase of ROS levels, responsible for cellular oxidative damage and inflammation involving the NF-κB pathway. When ROS concentration decreases following treatment with the K formulation, probably due to its direct scavenging ability and stimulating activity on cellular anti-oxidant systems, a reduction in the activation of NF-κB signaling occurs and, consequently, in the production of inflammatory cytokines and MMPs. In this scenario, Nrf2 expression appeared restored so that Nrf2-mediated anti-oxidant machinery resumes working correctly (i.e., SOD, CAT). However, even if the results show evidence that K formulation can exert a valuable anti-aging function on HDFs, it could be crucial to highlight the effect of each single component of the formulation and then assess possible additive and synergistic effects. Moreover, being the results obtained on dermal fibroblasts as a 2D model, additional research will be essential to evaluate the impact of K formulation on other cell types involved in the skin aging process (i.e., keratinocytes, melanocytes, macrophages), using innovative 3D skin models able to mimic the multi-layered structure of human skin [56]. Since the physiological cell-cell and cell-matrix interactions are properly recreated in 3D models, the obtained results will have a greater scientific soundness and be more correctly transferable to clinical practice. Despite these limitations, considering the central role of fibroblasts and oxidative stress in the skin aging process, our findings may well justify the clinical data recently reported by the group of Di Rosa et al. [30]. In this context, the finding on the ability of the K formulation to prevent or reduce the production of AGEs, whose generation and accumulation are considered detrimental in accelerating the aging process, in general, and skin aging, appears exciting. Thus, our results strongly support the K formulation’s anti-oxidant and anti-inflammatory features, which are essential requirements for an effective anti-aging treatment, as an oxidative imbalance closely linked to a strongly inflammatory condition rules the aged skin.

Thus, taking together, our findings suggest that the treatment with Karisma in the used experimental model had a reparative and regenerative effect in aged fibroblasts, leading to concentration-dependent recovery of physiological functions to levels not significantly different from non-aged controls.

## 5. Conclusions

In conclusion, the treatment with the poly-component Karisma formulation, due to its scavenging properties, could significantly reduce ROS levels back to the appropriate threshold, thus leading to the downregulation of NF-κB and, consequently, the shutdown of the inflammatory profile, including the reduction of the synthesis of pro-inflammatory cytokines and MMP-1 and -9. This condition could restore Nrf2 expression, which, in turn, can trigger the activation of cellular anti-oxidant systems (i.e., SOD, CAT). Overall, these findings help to clarify the mechanisms underlying the recovery of physiologic functions of dermal fibroblast cells previously reported by our group [29]. Our results also support recently published data on the clinical efficacy of the K formulation in vivo [30]. Moreover, considering that dermal fillers are generally very sensitive to the degradative action of oxidative stress [57,58], our results also appear interesting in this aspect. Indeed, in our aging model characterized by high levels of ROS, the formulation was able to reduce oxidative stress effectively and perform its anti-aging functions, implying that oxidative stress did not compromise its beneficial actions.

## Figures and Tables

**Table 1:** Primer sequences used in real-time PCR.

Target mRNA	Forward Primer	Reverse Primer
MMP-1	GCTAACAAATACTGGAGGTATGATG	GTCATGTGCTATCATTTTGGGA
MMP-9	CTGGACAGCCAGACACTAAAG	CTCGCGGCAAGTCTTCAGAG
GAPDH	TTGCCCTCAACGACCACTTT	TGGTCCAGGGGTCTTACTCC

## Data Availability

The datasets generated and analyzed during the current study are available from the corresponding authors upon reasonable request.
